# Distal bile duct carcinomas and pancreatic ductal adenocarcinomas: postulating a common tumor entity

**DOI:** 10.1002/cam4.566

**Published:** 2015-12-09

**Authors:** Rosa B. Schmuck, Cynthia V. de Carvalho‐Fischer, Christopher Neumann, Johann Pratschke, Marcus Bahra

**Affiliations:** ^1^ General, Visceral and Transplantation SurgeryCharité ‐ Universitätsmedizin BerlinBerlinGermany

**Keywords:** Bile duct neoplasm, cholangiocarcinoma, pancreatic neoplasm

## Abstract

The set definition of distal cholangiocarcinomas and adenocarcinomas of the pancreatic head is challenged by their close anatomical relation, similar growth pattern, and corresponding therapeutic outcome. They show a mutual development during embryologic organ formation and share phenotypic characteristics. This review will highlight the similarities with regard to the common origin of their primary organs, histopathological similarities, and modern clinical management. Thus, we propose to subsume those entities under a common superfamily.

## Introduction

The defining feature of a tumor has hitherto been its organ of origin, thus determining the treatment strategy that is most beneficial for patients suffering from a malignant disease. It follows from this concept that carcinomas of the distal bile duct (dCC) and ductal adenocarcinomas of the pancreas (PDAC) are defined as two independent tumor entities. This is in accordance with the current WHO classification of tumors, which distinguishes between tumors of the liver and intrahepatic bile ducts, tumors of the gallbladder and extrahepatic bile ducts, and tumors of the exocrine pancreas. Here, dCC is defined by the location of the main tumor mass only, not by the microscopic aspect 1. Given the spatial proximity of the duodenal curve, the pancreatic head, and the extrahepatic bile duct in the epigastric region, discriminating these single‐organ structures with accuracy reveals constitutional limitations. The intertwined anatomy of the proximal pancreatic duct and the distal bile duct (pervading the pancreatic head) gives ample reason to believe that both structures share more common features than previously believed. Moreover, both organs developed along similar embryological paths, and thus share numerous phenotypic characteristics. This hypothesis is corroborated by the fact that tumors of the distal bile duct and the pancreatic head also share the functional characteristics: a similar growth pattern, poor response to conventional chemotherapy, and a fairly unfavorable prognosis. We postulate that dCC and PDAC are a common tumor entity or should at least be subsumed under a common superfamily. Ampullary cancer, more particularly the pancreatobiliary subtype, should be considered as a part of this superfamily of tumors of the pancreatobiliary junction. The objective of this review is to define the common features as well as certain differences between these tumor entities, with regard to the embryonical development of the organ of origin, their diagnostic discrimination, histopathological and molecular similarities, and surgical and oncological treatment.

## The Embryologists View

Although biliary epithelial cells coat both the intrahepatic as well as the extrahepatic bile ducts, their developmental origins are distinct depending on their location in the biliary tree.

**Figure 1:**
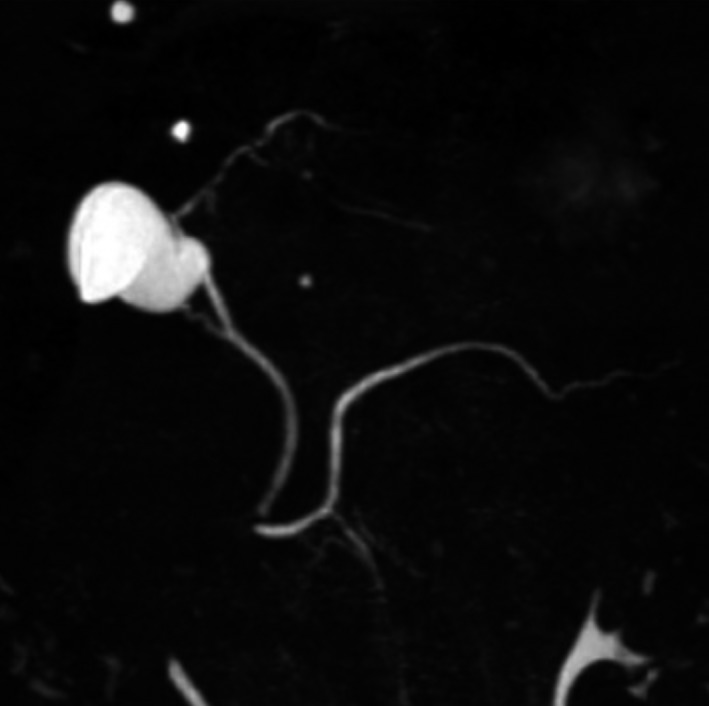
Pancreas divisum with main pancreatic duct and duct of Santorini in endoscopic retrograde cholangiopancreatography.

During the development of the gastrointestinal system, the endoderm forms the primitive gut. In the junction between the foregut and midgut that evolves into the duodenum, two outpunchings of the foregut form the so‐called ventral and the dorsal bud, which serve as the basis for the pancreas and parts of the bile duct system. The larger bud, located on the dorsal aspect of the primitive gut forms the cranial part of the pancreatic head, the body, and the tail of the pancreas including their corresponding ductal tree. The smaller ventral bud forms the caudal section of the pancreatic head, the uncinate process and the proximal part of the main pancreatic duct (Duct of Wirsung). The distal bile duct also has its origin in the ventral bud and temporarily forms a common channel with the proximal part of the main pancreatic duct. As the embryo develops, the ventral bud, containing the primitive proximal pancreatic duct and distal bile duct, rotates dorsally and fuses with the dorsal bud. The proximal part of the pancreatic duct system in the dorsal bud obliterates completely. In some cases this obliteration is incomplete and the mostly nonfunctional, accessory pancreatic duct (duct of Santorini) persists. The remaining ductules of the dorsal bud fuse with the ductal system of the ventral duct. Meanwhile, the distal bile duct and the pancreatic duct separate and remain connected solely at the duodenal conjunction, the so‐called ampulla of Vater 2, 3. If the rotation, and particularly the fusion of the ventral and dorsal duct are impaired, two separate pancreatic ducts remain and a so‐called *pancreas divisum* is formed (Fig. 1).

The hepatic diverticulum, which develops from the ventral foregut and remains attached to the abovementioned ventral bud, forms the basis for liver development. Mesenchymal cells surrounding the septum transversum induce progenitor cell differentiation into hepatoblasts, and stimulate the creation of the livers glandular structure. In the course of this development, biliary epithelial cells arise from hepatoblasts in the periportal layer. During the following phase of ductal plate remodeling, focal dilations of the aforementioned biliary epithelial cell precursors form bile ducts. Multiple defects in ductal plate remodeling lead to the formation of cysts rather than bile ducts, resulting in the so‐called Alagille syndrome 4.

In conclusion, the proximal pancreatic and the distal bile duct arise from common endodermal structures, thus reveal mutual characteristics in organ development and formation. In contrast, the pancreatic duct leading from central and caudal part of the pancreas originates from a separate endodermal portion. The intrahepatic bile duct system in contrast to the extrahepatic bile duct, takes its origin from periportal hepatoblasts in the hepatic diverticulum.

## PDAC and dCC: Analogies in Tumor Genesis

Intrahepatic cholangiocarcinomas (IH‐CC) are especially known for their histological diversity. Until recently, it was thought that this neoplasm is mostly likely derived from biliary epithelial cells, whereas it is now thought that this cancer can alternatively take its origin from hepatic progenitor cells. In opposition, extrahepatic cholangiocarcinoma (EH‐CC) arises from the biliary epithelium and the peribiliary glands 5. It is therefore of particular interest that a subset of pancreatic exocrine acini are physiologically interspersed within these same peribiliary glands 6. Three cell types have been identified in these acini: acinar cells with eosinophilic zymogen‐like granules, clear cells resembling centrioacinar cells and ductular elements. Additionally, peribiliary glands have been found to contain trypsin and amylase enzymes 7. A recent comparative study in transgenic mice as well as human tissue, focusing on precursors of PDAC underlined that PDACs originate from the ductal system and adjacent structures 8. It has been suggested that PDAC develops in the centroacinar–acinar compartment by acinar‐ductal metaplasia. Pancreatic intraepithelial neoplasia (PanIN) lesions have been shown to be the precursor lesions of PDAC 9. In transgenic mouse models, mucin‐producing ductular structures are frequently observed within the acinar parenchyma. These structures could represent an intermediate stage preceding the development of mouse PanIN. Another study analyzed the incidence and distribution of these ductular structures with and without mucinous metaplasia (mucinous tubular complexes/MCTs or tubular complexes/TCs). It could be demonstrated that the MCTs and TCs are common lesions in human pancreas. Furthermore, the expression of acinar markers, for example, trypsin was upheld in PanIN lesions 10. In conclusion, these results indicate that the origin of PDAC lies in the periphery of the ductal system. Therein lies a strong parallelism to the origin of EH‐CC, which also arises from the peribiliary and therefore periductal glands.

**Figure 2:**
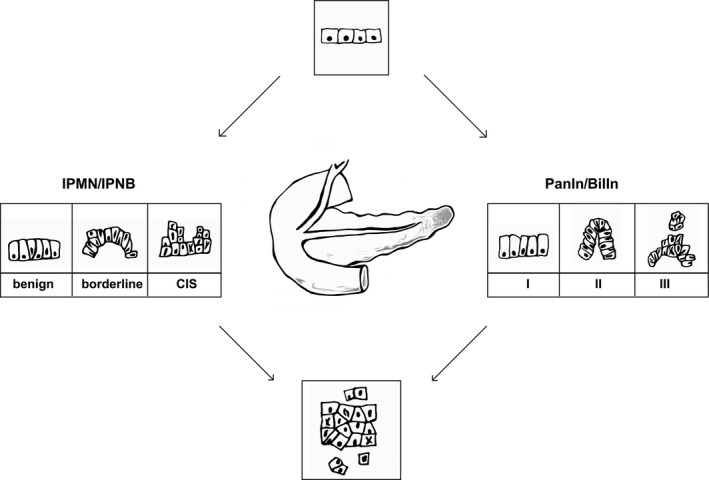
Biliary preneoplastic lesions and pancreatic precurser lesions share common characteristics. Both, PanINs (Intraepithelial neoplasia of the pancreas) and IPMNs (intraductal papillary mucinous neoplasms) have a corresponding counterpart in the biliary tract: BilINs (Biliary intraepithelial neoplasia) and IPNBs (intraductal papillary neoplasm of the biliary tract).

Intraepithelial neoplasia have been identified as premalignant lesions in several organs such as the pancreas (PanIN) or the prostate (PIN). Biliary intraepithelial neoplasia (BilIN) is assumed to be premalignant lesions of the bile duct system 11. BilINs are located both in the extrahepatic bile ducts and in the major intrahepatic bile ducts. By analogy to ductal carcinomas of the pancreas (PanIN 1 to PanIN 3), BilINs are classified into three grades 12. BilIN‐1 describes low‐grade dysplasia with mild cellular/nuclear atypia, BilIN‐2 is defined as intermediate grade whereas BilIN‐3 stands for high‐grade dysplasia and contains significant cellular and nuclear atypia. On account of the fact that BilIN‐3 corresponds to a carcinoma in situ*,* it considered as an overt cancer of the bile ducts. A comparison between BilIN and PanIN reveals impressive similarities. In conclusion, BilIN should be seen as a biliary counterpart of PanIN (Fig. 2).

Recently, intraductal papillary mucinous neoplasms (IPMN) of the pancreas were identified as precursor lesions with high malignant potential 13. Especially main duct IPMNs are associated with a high risk for transformation into intraductal papillary mucinous carcinomas (IPMC) 14. It is current consensus that two different precursor lesions in the pancreas have to be distinguished, namely PanINs and IPMNs. If one assumes that BilINs are the biliary counterpart of PanINs, is there also a biliary counterpart for IPMN? Biliary papillomas/papillomatosis may present suitable candidates, due to their role as premalignant lesions for invasive biliary carcinomas. Furthermore, biliary papillomas/papillomatosis and IPMN possess similar clinicopathological features 15. By virtue of this analogy, an alternative name was proposed for biliary papillomas/papillomatosis, namely the intraductal papillary neoplasm of the biliary tract (IPNB).

IPNBs are located in the extrahepatic bile duct (primarily hilar and distal), as well as in larger intrahepatic bile ducts. Furthermore, invasive IPNB lesions show elements from both tubular adenocarcinoma and mucinous carcinoma, which is also a well‐studied invasion pattern in IPMNs. In conclusion, biliary preneoplastic lesions and pancreatic precurser lesions share common characteristics. Both, PanINs and IPMNS have a corresponding counterpart in the biliary tract: BilINs and IPNBs (Fig. 2). Premalignant lesions congruent with those concepts have not yet been described for ampullary cancer. The Ampulla Vateri is the epithelial junction of the main pancreatic duct and the distal bile duct. Ampullary cancer may arise either from the intestinal epithelium or the pancreatobiliary ducts 16. The pancreatobiliary subtype shows a positive correlation with a higher rate of tissue invasion, lymph node metastasis, and a worse outcome, than the intestinal type 17. Thus, it has been hypothesized that the pancreatobiliary subtype should be seen as a very distal PDAC or dCC. In accordance with the aforementioned concept, PDAC and dCC and ampullary adenocarcinoma should be considered as a common tumor entity 16. Never the less, the focus of this review remains the comparison of PDAC and dCC subentities.

## Molecular Pattern: Differences and Analogies Between PDAC and EH‐CC

The two most commonly mutated genes in PDAC are p53 and KRAS. Activating KRAS mutations can be found in up to 95% of PDACs 18. It is of particular interest that KRAS mutations are present in 90% of early stage PDACs, as well as in premalignant lesions such as PanINs 19, 20. Through the analysis of KRAS mutations in biliary tract neoplasms and their corresponding premalignant lesions, Hsu et al. proved a similar process of carcinogenesis in the progression of BilINs to CCs 21. Moreover, a recent study reports that KRAS mutations are present in 61.1% of the ampullary cancers, but only in 15.2% of bile duct and 2.7% of gallbladder cancers 22.

One of the key players of physiological apoptotic response, tumor suppressor gene p53, is frequently subject to mutations in CC as well as PDAC. Khan et al. reported mutation rates of 20% to 61% for dCC 23, and another study determined that a low (0–30%) p53 expression showed a favorable prognostic factor in patients with resected dCC 24. In PDAC, p53 mutations can be found in up to 70% of primary, and in more than 50% of metastatic pancreatic cancers 25, 26, 27.

Unlike KRAS, p53 mutations seem to be a late event in the development of PanINs to PDAC 20, 28. Nevertheless, p53 has already been the subject of therapeutic approaches in PDAC. Transfer of the p53 gene via vectors has lead to an inhibition of tumor cells in vitro as well as in vivo 29, 30. It is of further note that mutant p53 stimulates chemoresistance against gemcitabine, and the reintroduction *(reactivation)* of p53 increases the cytotoxicity of gemcitabine 31, 32. Despite the aforementioned results, no attempt to evaluate p53 as a therapeutic target has been made in CC.

In recent years, miRNAs have emerged as diagnostic, as well as therapeutic targets in multiple cancers. miRNAs are small, noncoding RNAs that influence multiple physiological and pathological processes on the post‐transcriptional level. Furthermore, they have been identified as crucial players in carcinogenesis due to their role as both oncogenes and tumor suppressors. miR‐21 as well as let‐7a undergo an upregulation in PDAC and CC, 33 and may additionally modulate gemcitabine‐induced apoptosis, thus lending these molecules a potential therapeutic relevance 34. In a similar study, the targeted inhibition of miR‐21 via lentoviruses leads to a significant regression of PDAC tumor growth in vivo 35.

Despite these promising results, current literature does not enable a direct comparison of miRNA expression levels in PDAC and CCs. Furthermore, many projects do not distinguish between IH‐CCs and EH‐CCs, rendering a scientifically based conclusion on the similarities and difference between PDAC and dCCs impossible (Table 1) 36.

**Table 1:** Summary of similarities and differences in IH‐CC, EH‐CC, dCC, and PDAC

	IH‐CC	EH‐CC and dCC	PDAC	References
Developmental origin	Mesenchymal cells surrounding the septum transversum form small intrahepatic bile ducts	Ventral part of forgut forms main extrahepatic bile ducts	Ventral part of forgut forms mainpancreatic duct	2, 3, 4
Tumor genesis	Hepatocytes, hepatic progenitor cells or BECs	Ductal system or periductal glands	Ductal system or periductal glands	5, 6, 7, 8
Premalignant lesions	No corresponding lesions in IH‐CC	Biliary intraepithelial neoplasia (*BilIN*)	Pancreatic intraepithelial neoplasia (*PanIn*)	9, 10, 11, 12
Precursor lesions with malignant potential	No corresponding lesions in IH‐CC	Intraductal papillary mucinous neoplasms (*IPMN*)	Intraductal papillary neoplasm of the biliary tract (*IPNB*)	13, 14, 15
Molecular pattern	KRAS+/−	KRAS+	KRAS+	18, 19, 20, 21, 22, 23, 24, 25, 26, 27
	p53+/−	p53+	p53+	
Phenotype	CK20−	CK20+	CK20+	39, 40, 41, 42, 43, 46, 47, 48, 49, 50, 51, 52, 55, 56, 57
	MUC1+/−	MUC1+	MUC1+	
	MUC4+ (only larger bile ducts)	MUC4 +	MUC4+	
	S100P+ (only larger bile ducts)	S100P +	S100P+	
Surgery	Hemihepatectomy	PPPD/Kausch‐Whipple; extended hemihepatectomy for Klatskin‐Tumors	PPPD/Kausch‐Whipple	72, 73, 74, 75
Response to Chemotherapy	5‐FU+Gemcitabine−CapOx−Gemcitabine+cisplatin+nab‐Paclitaxel+/?	5‐FU+Gemcitabine+CapOx+Gemcitabine+cisplatin+nab‐Paclitaxel+/?	5‐FU+/−Gemcitabine+CapOx+Gemcitabine+cisplatin–nab‐Paclitaxel+	78, 79, 80, 81, 82, 83, 84, 85, 86, 87, 88, 89, 90, 91, 92, 93, 94, 95, 96, 97

## The Pathologists View: Are There Phenotypical Similarities Between PDAC and dCC?

The diagnosis of PDAC has hitherto been based on the identification of tumor markers such as CEA, CK19‐9, CK7, and CK20, in conjunction with its histological and clinical characteristics.

Cytokeratin (CK) 7 is a pancreatobiliary marker expressed in IH‐CC, EH‐CC, and pancreatic neoplasms 37, 38, 39, 40, 41, 42. Similarly, CEA and CK19‐9 are also expressed by all three tumor entities 16. Cytokeratin 20 shows a more nuanced expression, in that it revealed high expression in EH‐CCs and variable negative expression in IH‐CCs 39. In PDAC, CK20 expression was highest in tumors with a high number of reactive cells 40, despite an overall more common CK7+/CK20‐phenotype 40, 41, 42. Nevertheless, other studies suggest higher expression rates (65%) for CK20 in pancreatic cancer, and a plausible negative prognostic significance of the marker 43. Interestingly, the pancreatobiliary subtype of ampullary cancer lacks CK20 in majority of cases whereas the intestinal subtype shows a high CK20 expression 16, 44.

In conclusion, common tumor markers such as CK7, CEA, and CK19‐9 are unable to differentiate IH‐, EH‐CCs, and PDAC. Moreover, although CK20 shows a subtler expression prolife (low in IH‐CC, high in EH‐CC, and variable expression in PDAC), it demonstrates a high biological and methodical variability, rendering it an unreliable marker for pancreatobiliary cancers.

The inadequacies of current tumor marker panels have led to the exploration of alternative indicator molecules, among which proteins from the mucin and cadherin family have taken on a distinctive role. Such research could be valuable for identifying potential tumor subentities and further describing their clinical behavior, that is, drug response, overall survival, and rate of metastasis/invasion. Specific targeting of strongly expressed antigens could also lead to personalized therapeutic interventions.

Mucins (MUC) are high‐molecular, highly glycosylated proteins that form biological gels as a means of protecting epithelia from their external conditions. Furthermore, mucins play an important role in cell‐to‐cell signaling, and inducing immune reactions 45. A set of twenty different mucins has been identified in human epithelia, each glycosylated differently in order to carry out a specific function 46. Despite this biological variety, this comparative analysis will focus on solely MUC1 and MUC4 as potential tumor markers.

MUC1 has been identified in both pancreatic and so‐called “mucin‐producing” cholangiocarcinomas derived from the large bile ducts of the liver 47, 48, 49, 50. Tamada et al. revealed that while MUC1 is not expressed in normal epithelia of the bile duct, it can be found in 87% of EH‐CC tumors analyzed (*n* = 70) 51. More importantly, MUC1 showed an analogous expression pattern in biliary intraepithelial and pancreatic intraepithelial neoplasms 52. Furthermore, MUC1 may promote cell adhesion in foreign tissues, and thus allows for PDAC invasion 53. In comparison to the above mentioned MUC1‐expressing tumors, mixed‐type CCs, and cholangiocarcinomas (deriving from hepatic progenitor cells rather than cholangiocytes) only produce negligible amounts of MUC1 47.

Interestingly Remmers et al. showed that MUC1's expression and glycosylation pattern transformed from normal tissue to premalignant and cancerous tissues of the pancreas 46. This study detailed that unglycolyzed MUC1 was highest in metastatic tissue, whereas “T‐MUC1” (a specifically glycolyzed form of the protein) expression was lowest in normal tissue. Hence, MUC1 could potentially be used to map the progression of normal to malignant tissue in both pancreatic and extrahepatic biliary cancer.

Although MUC1 functions as a membrane receptor, MUC4 may bind to the ErbB2 receptor, possibly increasing p27 Expression 54. Several studies have shown that while MUC4 is not present in normal liver or pancreatic tissue, it is present in PDAC, EH‐CC, and IH‐CC derived from the larger bile ducts 46, 51, 55, 56, 57. More importantly, it has been identified as a negative prognostic factor for survival in all three tumor types correlating with higher metastatic and lymphatic invasion 55, 56, 57. In IPMN's of the pancreas, MUC4 may enable a differentiation between malignancies of the intestinal type and the gastric type 56. Unfortunately, data on MUC4 expression in mixed‐type CCs is scant.

Proteins from the cadherin family have also recently come into the spotlight as potential indicator substances for gastrointestinal neoplasms. Cadherins play an important role in cell adhesion and cell communication, both as receptors and ligands within signaling pathways. Additionally, it has been postulated that during epithelial–mesenchymal transition (EMT) a so‐called “cadherin switch” comes into effect, in which E‐Cadherin, typically expressed in epithelia, is replaced by N‐Cadherin, found mostly in mesenchymal tissue 58. E‐Cadherin has proved to be a protective factor in both CC and PDAC 59. Nitta and Mitsuhashi et al. recently showed that N‐Cadherin was a negative prognostic factor for survival in EH‐CC, a finding confirmed by Araki et al. 60, 61. Elevated expression of N‐Cadherin in IH‐CC was also linked to aggressive behavior with increased metastasis and invasion 62. However, a recent study was able to show that N‐Cadherin expression was significantly lower in metastatic liver PDAC than in CCs, possibly enabling a differentiation of these tumor subtypes in conjunction with other markers 63. Unfortunately, this study did not discriminate between subentities of cholangiocarcinoma.

Finally, S100p encodes a family of proteins responsible for regulating differentiation and proliferation. Ali et al. have identified this protein as a highly specific and sensitive marker for pancreatobiliary tumors, achieving 100% specificity and 83% sensitivity on its own 64. By means of a histological panel including S100p, MUC1, KOC, and mesothelin, the diagnosis of pancreatobiliary tumors was achieved to nearly 100% specificity and sensitivity, in cytological specimens sampled through endoscopic retrograde cholangiopancreatography (ERCP). Other studies confirm high S100p expression in PDAC and EH‐CC, in contrast to infrequent expression in normal pancreatobiliary tissue 65. A recent study showed that S100P staining identified IH‐CCs with bile duct morphology (a.k.a derived from the larger ducts of the biliary tree) to have a similar expression profile (CEA+, CK19‐9+, MUC2+, and more likely N‐Cadherin negative) to EH‐CC and PDAC 66. S100p negative tumors showed less of a bile duct morphology and were more likely to be N‐Cadherin negative, Liau et al. confirmed a higher expression of S100p in IH‐CC of bile duct morphology.

Although EH‐CC and PDAC show many similarities concerning the expression of MUC1 and S100P, IH‐CC derived from the small bile ducts seem to have a different phenotype. MUC4, N‐Cadherin may also prove to be useful markers, given further study. In conclusion, standardized immunohistochemical panels including atypical marker proteins should be further evaluated in order to improve the accuracy of IH‐CC and EH‐CC diagnosis.

## Assigning a Diagnosis: Imaging Strategies for PDACs and dCCs in Light of Common Features and Anatomical Difficulties

Diagnostic methods for discriminating cancers of the pancreatic head and the distal bile duct often overlap. In this chapter, we aim to highlight common features and challenges in the discernment of the abovementioned entities.

**Figure 3:**
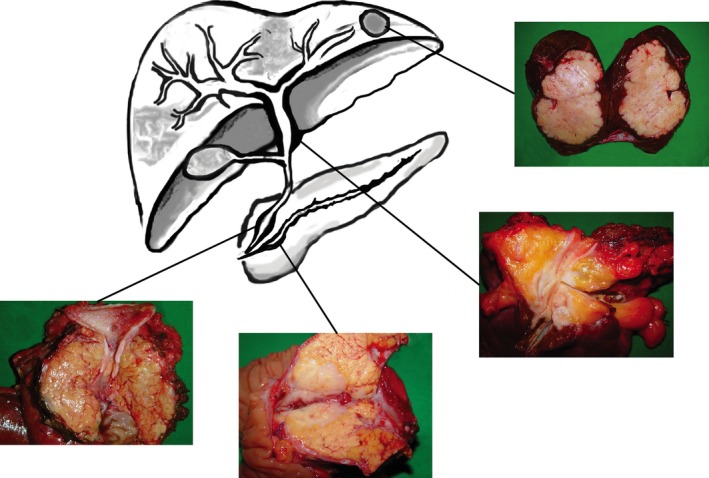
Tumors are classified according to their location (from top to bottom): intrahepatic cholangiocarcinoma (IH‐CC), hilar cholangiocarcinoma, distal cholangiocarcinoma (dCC), and adenocarcinoma of the pancreas (PDAC).

The early detection of the tumor is crucial for the prognosis of PDAC and dCC patients. Both neoplasms arise from ductal epithelia, and therefore tend to grow in longitudinal alignment with the ductal system during the early stages of tumor formation. It is due to this mechanism that mass formation occurs mostly in the later stages of the tumor development, impeding an early, and thus potentially curative, image‐based diagnosis. In contrast, IH‐CCs show a distinct growth pattern (including concentric growth and mass formation), thus facilitating tumor detection in an abdominal ultrasound (US), computed tomography (CT), and/or magnetic resonance imaging (MRI) (Fig. 3). Therefore, the longitudinal rather than concentric growth pattern of PDAC and dCC severely restricts image‐based screening, due to their limited sensitivity. Through a comparison of CT, MRI, magnetic resonance cholangiopancreatography (MRCP), and the endoscopic ultrasound (EUS), the CASPS3 study showed that the best visualization of small pancreatic lesions could be achieved by EUS and MRCP 67. This result may be explained by the occurrence of neoplastic obstruction of the ductal system, which can occur in early stages of PDAC and dCC progression when the tumor itself is not yet detectable via radiological assessment**.** An additional benefit of the EUS is the possibility to take samples via fine needle aspiration or brush cytology. A sufficiently large tumor sample can discriminate between benign and malignant lesions in selected cases. Studies on intraductal endoscopic ultrasound (IEUS) took the EUS approach to dCC and PDAC identification a step further, and demonstrated that the diagnosis of bile duct strictures and lesions of the pancreatic head can be improved by the assessment of the ductal wall and its surrounding structures 68, 69, 70. In comparison to IH‐CCs and lesions of the caudal pancreas, PDACs and dCCs can be easily assessed via ERCP, EUS, and IEUS. Furthermore, the early diagnosis of the latter tumor entities is mostly based on the visualization of the concomitant cholestasis. A large portion of PDACs, dCCs as well as ampullary cancers clinical symptoms, result from cholestasis due to neoplastic obstruction. Therefore, tumors with a high cholestatic effect are often diagnosed at an earlier stage due to their prompt onset of symptoms. The same explanation may clarify the slightly higher survival of dCC as compared to PDAC: tumors located in the corpus or cauda of the pancreas will not cause cholestasis in the early stages of the condition. Another reason for this finding might be the fact that some studies include ampullary cancer (a neoplasm with an overall more favorable prognosis) into prognosis estimation of dCC 71.

Due to the contiguity of PDACs and dCCs, the discrimination of these neoplasms can be particularly challenging. A clear anatomical attribution can be especially difficult in later tumor stages, when malignant growth involves neighboring structures. Applying the hypothesis of a common superfamily for those tumor entities, a distinction according to the organ of origin would be obsolete. The common tumor entity approach becomes a depiction of clinical reality in that the only possibility for curative treatment, that is a radical surgical resection, is congruent concerning the surgical technique for PDAC and dCC.

## The Surgeons Point of View: Surgical Treatment Options in PDAC and CCs

Radical surgical resection with a microscopic tumor‐free resection margin (R0), is the only curative option for PDAC and CC 72. The guiding principle behind radical resection applies to the whole range of tumors of the pancreatobiliary system, regardless of their location. Thus, the procedure of choice for hilar cholangiocarcinoma (Klatskin tumor) is an extended hemihepatectomy, with en bloc extrahepatic bile duct resection. This procedure has a clear advantage regarding the local recurrence rate and overall survival time, as compared to alternative strategies. Neuhaus et al. described a 1‐, 3‐, and 5‐year survival rate after hilar en bloc resection, which included the removal of the portal vein bifurcation, of 87%, 70%, and 58%, respectively. Survival rates after conventional major hepatectomy were significantly lower with 1‐, 3‐, and 5‐year survival rates of 79%, 40%, and 29%, respectively 73. Due to the spatial proximity of the duodenum, the pancreatic head, the extrahepatic bile duct, and their overlapping blood supply, curative resections of both PDAC and dCC require a pancreatoduodenectomy, preferably under preservation of the pylorus (PPPD). In selected cases of ampullary cancer, a local resection via transduodenal ampullectomy or an endoscopic resection can be considered 74. As a result of the longitudinal spread of the tumor in the bile duct wall, an additional hemihepatectomy has to be considered in selected cases in order to achieve an R0 resection 75. From a surgeon's point of view, a suspected malignant lesion located in the pancreatic head – regardless of whether it arises from the pancreatic or the bile duct – needs to be removed in toto using an identical surgical technique.

## The Oncologists Perspective: How Do Pancreatobiliary Tumors Respond to Conventional Adjuvant Chemotherapy?

There are various therapeutic options for pancreatobiliary cancers, namely PDAC, IH‐CC, and EH‐ICC 76, 77. Nevertheless, most patients diagnosed with these types of cancer have a low survival rate and die within the first one to three years of diagnosis with or without chemotherapeutic treatment 78, 79, 80.

Common therapeutic agents for pancreatobiliary cancers include antimetabolites such as 5‐fluorouracil (5‐FU) 80, 81 and capecitabine 82, 83, 84, nucleoside analogs such as gemcitabine 85, 86, 87, 88, 89, 90, platinum analogs such as oxaliplatin, 82, 83, 84 and cisplatin 91, 92. In recent studies, chemicals known as taxenes in form of *nab*‐paclitaxel, a 130‐nm albumin‐bound formulation of paclitaxel particles, have been evaluated to this end 93.

While 5‐FU is not the first preference for adjuvant chemotherapy of PDAC and is only reported to have minimal effects on overall survival 81, it has a much bigger impact on IH‐CC and EH‐CC. A retrospective study by Yoshitda et al. including 26 patients with distal bile duct cancer after pancreaticoduodenectomy, reports a 5‐year‐survival rate of 56% with adjuvant chemotherapy compared to 27% for untreated patients 80. Similarly, another study carried out with a collective of 35 patients with intraductal papillary peripheral cholangiocarcinoma, accounts for a 5‐year‐survival rate of 33.3% for treated and 10.8% for untreated patients 79.

Gemcitabine, on the other hand, has been used very successfully in adjuvant chemotherapy to increase the overall survival rates of patients suffering from PDAC, exemplary shown within the scope of the CONKO Study by Sinn et al. 94, 95.

A multicentered, randomized, phase III, clinical trial compared the effects of gemcitabine with 5‐FU for pancreatic carcinoma in first‐line palliative setting. The study concluded that the clinical benefits (23.8% vs. 4.8%) as well as the 12‐month survival rate (18% vs. 2%) were both significantly greater for gemcitabine than for 5‐FU, respectively 90.

A similar therapeutic effect has been reported for Gemcitabine in EH‐CC. According to Murakami et al. the 5‐year‐survival rate of patients who received adjuvant chemotherapy with gemcitabine after extended surgery for hilar cholangiocarcinoma, was noticeably higher (57%) compared to those who did not receive any postoperative treatment (23%) 85. For IH‐CC, however, Gemcitabine is reported to have minimal effect 86. Thus, the clinical response to gemcitabine is similar for PDAC and EH‐CC, in contrast to IH‐CC.

Several alternative treatment protocols for pancreatobiliary cancers involve platinum‐based therapeutic agents. The combination therapy of capecitabine and oxaliplatin (CapOx), for example, offers a different treatment for PDAC with similar outcomes to gemcitabine‐related regimes 84. A prospective, multicentre, phase II trial by Nehls et al. analyzed the response of 47 patients with EH‐CC with respect to CapOx treatment, and compared the results with 18 patients with IH‐CC who received the same chemotherapy. The response rate of the EH‐CC collective was reported to be 27%, while there were no objective responses among the IH‐CC patients. Additionally, 49% of the EH‐CC had a stable disease after treatment compared to 33% for the IH‐CC 83.

On the other hand, reports suggest that a gemcitabine and cisplatin combination therapy leads to different clinical responses. According to an extensive phase II study by Valle et al. both EH‐CC and IH‐CC have a better clinical response to the above mentioned combination therapy, as compared to a gemcitabine monotherapy 78. In PDAC, however, this combination therapy only shows moderate activity, highlighted by a low response rate of 11% 88. Hence, while PDAC and E‐CC respond to CapOx analogically, the response is quite different for a gemcitabine and cisplatin combination therapy.

Recently, the use of nab‐Paclitaxel in conjunction with gemcitabine for treating PDAC has/come into the spotlight. A contemporary phase III study compared a collective of 430 patients with PDAC on a gemcitabine monotherapy with 431 patients who received a combination of gemcitabine and nab‐Paclitaxel. The treatment resulted in a higher median overall survival for the combination therapy collective (8.5 vs. 6.7 months), despite higher rates of peripheral neuropathy and myelosuppression 96. However, there have been no extensive clinical trials in cholangiocarcinoma populations so far. A recent study by Kolinsky et al. examined the therapeutic effect of nab‐Paclitaxel monotherapy for two patients with cholangiocarcinoma and suggests that nab‐Paclitaxel does indeed have therapeutic activity 97. Considering the similarities between PDAC and EH‐CC relative to their therapeutic response, as outlined in this section, future studies examining the therapeutic activity of a nab‐Paclitaxel and gemcitabine combination in EH‐CC are more than justified. This is of particular significance as chemotherapy algorithms of dCC and PDAC currently diverge, particularly for advanced disease.

## Conclusion: New Thoughts and Concepts on Defining Pancreatobiliary Cancers

Carcinomas of the pancreatic head and dCCs share a wide range of common features. Both tumors possess a common embryologic origin, a marked anatomical overlap of the primary organ tissue, and several phenotypic analogies. All of these factors explain the difficulties in discriminating both tumor entities through imaging studies, and the exigency for radical surgical resection. Finally, similarities in the therapeutic outcomes of PDAC and extrahepatic bile duct cancer suggest strong behavioral analogies. Summarily, a new and unified superfamily for those tumor entities of the pancreatobiliary junction should be considered to more accurately define these neoplasms. We consider the definition of such a superfamily more adequate than the simple categorization of IH‐CC and dCC as cholangiocarcinomas.

## Conflict of Interest

None declared.
